# Evaluating the potential of Unmanned Aerial Systems for mapping weeds at field scales: a case study with *Alopecurus myosuroides*

**DOI:** 10.1111/wre.12275

**Published:** 2017-11-16

**Authors:** J P T Lambert, H L Hicks, D Z Childs, R P Freckleton

**Affiliations:** ^1^ Department of Animal & Plant Science University of Sheffield Sheffield UK; ^2^ CSIC Madrid Spain

**Keywords:** black‐grass, distribution, drones, modelling, precision agriculture, site‐specific weed management, wheat

## Abstract

Mapping weed densities within crops has conventionally been achieved either by detailed ecological monitoring or by field walking, both of which are time‐consuming and expensive. Recent advances have resulted in increased interest in using Unmanned Aerial Systems (UAS) to map fields, aiming to reduce labour costs and increase the spatial extent of coverage. However, adoption of this technology ideally requires that mapping can be undertaken automatically and without the need for extensive ground‐truthing. This approach has not been validated at large scale using UAS‐derived imagery in combination with extensive ground‐truth data. We tested the capability of UAS for mapping a grass weed, *Alopecurus myosuroides*, in wheat crops. We addressed two questions: (i) can imagery accurately measure densities of weeds within fields and (ii) can aerial imagery of a field be used to estimate the densities of weeds based on statistical models developed in other locations? We recorded aerial imagery from 26 fields using a UAS. Images were generated using both RGB and R_mod_ (R_mod_ 670–750 nm) spectral bands. Ground‐truth data on weed densities were collected simultaneously with the aerial imagery. We combined these data to produce statistical models that (i) correlated ground‐truth weed densities with image intensity and (ii) forecast weed densities in other fields. We show that weed densities correlated with image intensity, particularly R_mod_ image data. However, results were mixed in terms of out of sample prediction from field‐to‐field. We highlight the difficulties with transferring models and we discuss the challenges for automated weed mapping using UAS technology.

## Introduction

One of the problems with managing weed populations is that weeds are non‐uniformly distributed at almost every spatial scale at which we study them. Weeds are undoubtedly patchily distributed within fields (Wilson & Brain, 1991; Nordmeyer, 2006). At the higher scales of fields, farms and landscapes, there can also be considerable variations in weed abundance (Thornton *et al*., 1990; Gabriel *et al*., 2005). Indeed, even at the national scale, some regions contain more weeds than others (Marshall, 2009). Such variations reflect the combined imprint of environment and management history (Fried *et al*., 2008). This non‐uniform distribution of weeds has long been recognised and, for over a century, attempts have been made to understand the factors that contribute to variation in weed distributions; for example, as long ago as 1913, Brenchley (1913) attempted to understand how soil and management contribute to variation in the occurrence of weed species in the United Kingdom.

Understanding the distributions of weeds requires that they are monitored. Monitoring of weed populations has typically focused on small‐scale detailed studies. For example, a literature search focusing on weed surveys prior to 2008 showed that 84% of all previous field plots were smaller than 1 m^2^ (Queenborough *et al*., 2011). Moreover, monitoring effort is typically limited in terms of the number of observers available, so that most sampling protocols sacrifice spatial scale for intensity. This means that effective sampling areas may be relatively small; for instance, in one of our previous demographic studies, we estimate that the sampled area was only 3% of the total experimental area (total experimental area = 36 × 48 = 1728 m^2^; monitored area = 48 m^2^; Lintell Smith *et al*., 1999). Limited sampling of this sort presents many issues, because systems can vary dramatically both spatially and temporally (Craufurd & Wheeler, 2009).

Large‐scale mapping has been undertaken to build up pictures of weed distributions at regional and landscape scales (Lawrence *et al*., 2006; Barnett *et al*., 2007; Cuneo *et al*., 2009). These analyses are usually based on coarse estimates of weed abundance. In the coarsest form, there are atlas measurements of occurrence at a scale as large as 10 × 10 km (Preston *et al*., 2002). Even at this scale, data are useful in analysing large‐scale geographical drivers of occurrence such as climate (Storkey *et al*., 2014). Field‐scale estimates of occurrence (presence/absence) or prevalence (density) have also been used to build up large‐scale pictures of the abundance of weeds (Joseph *et al*., 2006). Such data are extremely valuable in generating insights into the factors that drive weed abundance (Westbury *et al*., 2008; Henckel *et al*., 2015).

Mapping weed densities is thus a trade‐off between precision and extent; fine‐scale ecological monitoring generates detailed data on small scales, while large‐scale surveys generate coarse data across large extents. To bridge this gap, Queenborough *et al*. (2011) developed density‐structured monitoring approaches for arable weeds. This approach generates field‐scale maps of weed distributions. Within‐field mapping is relatively coarse (a 5‐point scale for assigning density states within large plots of size 20 × 20 m), but the approach is readily up‐scaled to hundreds of fields during a field season for a small team (e.g. 2 or 3 observers). Based on readily available resources (i.e. field walking/monitoring in small teams), this represents a compromise approach that generates large numbers of within‐field maps at among‐farm farm and regional scales. Data from such monitoring can be used to parametrise predictive ecological models (Freckleton *et al*., 2011) and henceforth be useful in solving a key problem that many models face, lack of comprehensive data (Tredennick *et al*., 2017).

Measuring weeds in an agricultural setting is undoubtedly useful for the monitoring and management of farm systems (Huang & Asner, 2009), but arguably limited by the trade‐off between precision and extent. However, recent technological advances have resulted in a step change in the potential to collect detailed ecological data at large scales. Unmanned Aerial Systems (UAS) are flying robots that can collect varied data, including colour and hyperspectral imagery allowing vegetation indices to be constructed, as well as environmental data (Nonami, 2007; Torres‐Sánchez *et al*., 2014). Prior to the introduction of UAS, satellites and manned aircraft were the only way of capturing aerial imagery of landscapes, with numerous applications in the monitoring and management of ecological systems (Kerr & Ostrovsky, 2003; Pettorelli *et al*., 2005). There have been attempts to use imagery generated by such platforms to map weeds on the field scale, but poor resolution of the imagery has previously limited its application (Lamb & Brown, 2001; Thorp & Tian, 2004).

A typical hobby grade UAS will have a pixel resolution of 2.8 cm pixel^−1^ when flown at 100 m altitude, flight time of 25 min and cost around €1000, therefore providing high resolution and low‐cost imagery for small survey areas. Compared with field walking and conventional ecological monitoring techniques, this technology offers considerable potential for addressing the trade‐off between resolution and extent. Consequently, there has been a substantial increase in interest in the use of UAS for mapping in the precision agriculture sector (Zhang & Kovacs, 2012).

Although UAS offer great potential to produce detailed data over large spatial extents, ultimately, they will be useful in research and management only if they can be shown to generate large amounts of reliable data. There have been attempts to use remote sensing to map populations in detail (Huang & Asner, 2009), but they have been limited in spatial and temporal scales (López‐Granados, 2011; Rasmussen *et al*., 2013). Nevertheless, there is significant commercial interest in the applications of UAS in agriculture. However, this interest has largely not translated beyond a proof of principle with some of the commercial ventures over promising, under delivering and subsequently failing (Catapult Satellite Applications, 2016).

Ultimately, for UAS‐derived imagery to be useful in weed monitoring, several conditions must be fulfilled. First, most importantly, it must be shown that imagery correlates closely with weed density. This is an obvious requisite for this technology to be practically useful. However, this is very difficult to test because to do so requires density data from many fields at fine spatial resolution to ground truth the imagery from UAS. As noted above, such data are difficult to acquire. Second, especially for management applications, the pipeline of data processing from image capture to weed density maps should include as few steps as possible. This is so that the technology is readily useable. Third, the platform and imagery should ideally be robust to variations between fields and observation conditions, so that minimal tuning or subjective interpretation by analysts is required. These conditions can be relaxed to varying degrees if additional local and context‐specific data are available. For example, variations in observation conditions (e.g. weather, light, soil, crop colour) can be accommodated by collecting ancillary data for calibration. However, this requires extra steps in data collection and analysis that may prove challenging or impractical in many applications. To date, although UAS are increasingly being used in field situations, the answers to these questions are largely unknown.

In this paper, we explore the potential for simple inexpensive UAS to acquire images that can be used in weed mapping. We focus on the use of readily available ‘off‐the‐shelf’ systems that can be used by researchers, agronomists and farm managers for quantitative analysis of weed distributions. The first major question we address is whether imagery from such platforms is capable of measuring weed densities? To do this we combine imagery from UAS with an extensive data set on weed populations across 26 fields. The second question is whether we can use models transferably across fields? We address this by developing statistical models that relate imagery and weed densities for one field and asking whether these accurately transfer to other sites. We show that in principle UAS‐derived imagery closely relates to weed densities. However, we highlight various challenges in automating the collection and analysis of data.

## Materials and methods

### Study system

The weed *Alopecurus myosuroides* Huds. (black‐grass) in winter wheat (*Triticum aestivum* L.) was chosen as a study system. This weed species has significant economic impacts on crop yields and is prevalent throughout northern Europe (Twining & Clarke, 2009). Black‐grass has been shown to significantly reduce yields when present (Blair *et al*., 1999) and infests approximately 70% of fields in the United Kingdom (Black‐Grass Research Initiative, BGRI unpubl. obs.).

We selected study sites that included both large and small farm sizes and differing crop rotations within each of the following five geographical regions in the United Kingdom: Oxfordshire, Bedfordshire, Norfolk, Lincoln and Yorkshire. Farm size varied from 80 to 3000 ha. Crop rotations varied from continuous cropping of winter wheat, to an 8‐crop rotation.

*Alopecurus myosuroides* populations were censused from the 1st of June 2015 to the 27th of July 2015, during which time, the weeds were mature and visually distinct, corresponding to 87–89 on the BBCH scale respectively (Lancahsire *et al*., 1991). In this period, 26 fields were surveyed across the five regions. This sample of 26 fields is by far the largest data set on within‐field weed distributions to have been used to assess the effectiveness of UAS technology. However, not all 26 georectified images were suitable for full analysis, due to poor data quality, resulting in 18 full fields suitable for full analysis.

### Weed population monitoring

We used the density‐structured approach (Taylor & Hastings, 1998) implemented by Freckleton *et al*. (2011) and Queenborough *et al*. (2011), in which five discrete density states (absent, low, medium, high, very high) were used to estimate *A. myosuroides* plant numbers. These discrete density‐structured observations have been shown to be representative of counts of plants (Freckleton *et al*., 2011; Queenborough *et al*., 2011). The advantage of the density‐structured approach over individual plant head counts is that it allows populations to be estimated very rapidly, permitting data to be collected at far greater spatial extent while also reducing fieldwork costs. Existing research suggests that misclassification between observers of density states is negligible (Collett, 2002).

**Figure 1:**
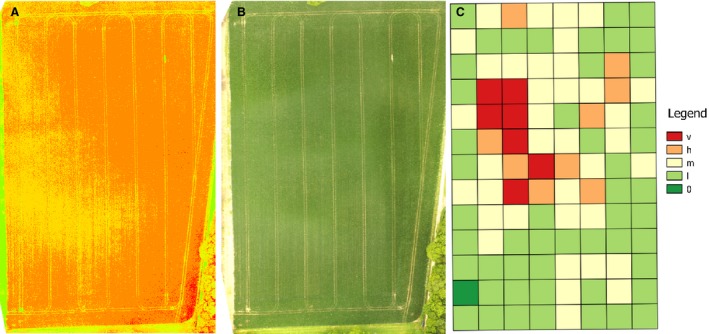
For illustrative purposes, this field was flown twice, and the camera was changed for each flight. With (A) greyscale colour enhanced R_mod_ and (B) RGB. This allows for side by side visual comparison of the image data, with the same underlying level of black‐grass, (C) overlay of the ground‐truthed observed density states. The legend corresponds to the accompanying density states that were recorded, ranging from very high (v) to absent (0).

Plots were 20 × 20 m, which is a convenient scale for monitoring(Queenborough *et al*., 2011). Surveys were performed by a team of three trained observers and the outcome of surveying on each field is a grid of density state measurements of the whole field (see Fig. 1 for an example). The five density states were assigned using the quartiles of densities determined in the Farm Scale Evaluation of GM crop trials (Heard *et al*., 2003). The five density states counted *A. myosuroides* plants per 20 m^2^ in bands of 0, 1–160, 161–450, 451–1450 and 1451+ respectively for absent, low, medium, high and very high‐density state observations.

### Collection of UAS images

To collect the UAS imagery data, we used a commercially available DJI Phantom 2 (Austin, 2010). Two cameras were used to collect images. Firstly, a modified GoPro Hero3 (‘GoPro Official Website – Capture + share your world − HERO3 +’. https://gopro.com/update/hero3_plus. Last accessed 24 February 2017) with a filter (https://event38.com/product/custom-ngb-filter-glass-for-diy-camera-conversion/) was used to capture modified colour aerial images and a 16.5 mm focal length, non‐fisheye lens was installed to reduce the image distortion (R_mod_GB: blue, B: 390–520 nm; green, G: 470–570 nm; red‐edge, R_mod_ 670–750 nm). Such images have been shown to be useful for mapping in an agricultural context (De Castro *et al*., 2015). Secondly, a Canon s100 (‘Canon PowerShot S100 Black Refurbished | Canon Online Store’. https://shop.usa.canon.com/shop/en/catalog/powershot-s100-black-refurbished. Last accessed 24 February 2017) was used to provide RGB images with focal length set to 24 mm. Spectral data can be found via the respective online sources. The images were stored in RAW format, and the cameras were triggered to capture images every 1.5 s via software control. White balance was set using a calibration card prior to each flight. The flights were flown autonomously in a grid pattern that used a 60% side and front overlap at a height of 100 m, this ensured optimal coverage of the target (Ballesteros *et al*., 2014). The average area covered over the 30 flights was 5.32 ha, an average GSD of 3.2 cm pixel^−1^ and an average flight time of 11 min.

### Data processing: image stitching

Individually, each image represents a limited view of the field. For field‐scale analysis, it is necessary to combine these subsamples into one image of high quality. We used a commercial desktop solution for this Agisoft (‘Agisoft PhotoScan’. http://www.agisoft.com/. Last accessed 1 February 2017). We then cropped the UAS imagery to the extents of the accompanying density state grids using the georeferenced orthomosaics on a field‐by‐field basis. We manually applied a soil thresholding mask, to cut out pixels that were observed to be soil on a field‐by‐field basis to remove the pixels of soil that are present in the tramlines or patches of bare ground in the field that could introduce a bias. This was performed in imageJ (‘ImageJ – RSB Home Page’. 2016. <https://imagej.nih.gov/ij/last accessed 25 October 2016) by visual inspection of the amount of bare soil visible in each image. We then combined the data sets, so that every pixel had their respective three spectral band values, a location in geospace and an observed density state which was dependent on its location within the field.

### Data analysis

#### Analysing correlations between weed surveys and imagery

The objective of the first set of analyses was to assess the ability of the mean pixel values of the 20 × 20 m plots for the respective spectral bands to capture explain variation in density states. A series of multiple linear regression models were fitted and then used to predict density states. A least squares model was fitted to the RGB data set using the spectral bands red, green and blue as the predictors and observed density state as the response variable. A second regression model for R_mod_ used the spectral bands red‐edge (R_mod_), green and blue as predictors, and observed density state as the response variable.

#### Testing predictive performance of images

The second set of analyses was designed to test predictive performance of statistical models fitted to imagery. We used a random forest classifier to evaluate the spectral data's ability to discern weed densities. A random forest model is an ensemble learning method that utilises classification and regression tree (CART) analysis (Breiman, 2001). The model was applied in two ways: (i) to predict the presence/absence of black‐grass and (ii) to discriminate between areas of high and very high *A. myosuroides* observations. We used the same spectral bands as the linear models for the respective data sets and fitted the random forest model with 32 000 trees. The spectral data sets were split into training and testing data at the 20 × 20 m scale, with the training data being used to build a random forest model and the testing data being used for predicting against. The data were split 80/20 respectively.

Area under the curve (AUC) and accuracy (ACC) were used as metrics to test the ability of the random forest model to predict the presence/absence of *A. myosuroides*. AUC is a measure of the area under a ROC (receiver operating characteristic) curve and is an alternative measure of goodness of fit. ACC is equal to the probability that a classifier will rank a randomly chosen positive instance higher than a randomly chosen negative one (Fawcett, 2006). This metric is important for assessing the predictive ability of the models.

#### Field‐to‐field predictions

The aim of this analysis was to measure the predictive performance of models by testing the extent to which a model developed in one field could be used to predict densities of weeds in other fields. We selected the R_mod_ data set for further analysis as this generated the best correlations between observed and fitted density from the linear regression models. To test the field‐to‐field predictive ability, we fitted a cubist model for each of the eight individual fields for which R_mod_ data were available (Table 1). Cubist models are rule‐based models that are created in a similar way to the random forest models but the terminal leaves contain linear regression models (Quinlan, 1992), thus allowing comparison to the initial analysis. Cubist models were chosen as they provide an ensemble classifier approach, resulting in an average prediction for the ensemble, as opposed to the single snapshot of the previous models. These models were constructed using all the data for each individual field and then used to predict the density states of the remaining fields. We assessed the performance of these models by recording the correlations between the predictions and the independent ground‐truthed observations.

**Table 1:** Explanatory power of imagery acquired by unmanned aerial systems to describe weed densities within fields

	Linear model			Random forest			
Field number	*R* ^2^	*P*‐value	d.f.	P/A AUC	P/A Acc	H/VH AUC	H/VH Acc
RGB							
1	0.1568	0.0015	134	0.9140	0.8390	N/A	N/A
2	0.0344	0.4195	200	0.4354	0.4354	N/A	N/A
3	0.0308	0.8234	113	0.5167	0.5069	N/A	N/A
4	0.1670	0.0013	127	0.6923	0.5618	N/A	N/A
5	0.1549	0.0000	234	0.8357	0.6027	0.3333	0.8165
6	0.1305	0.0135	124	0.7452	0.5707	N/A	N/A
7	0.0836	0.0126	202	0.8743	0.6781	0.7598	0.5849
8	0.0270	0.6304	189	0.5654	0.5319	N/A	N/A
9	0.4555	0.0000	94	0.8397	0.8094	N/A	N/A
Overall	0.2937	<2.2E‐16	1481	0.8828	0.6827	0.9073	0.8658
R_mod_							
10	0.0596	0.1127	187	0.5807	0.5215	N/A	N/A
11	0.2533	0.0000	128	0.9281	0.6238	0.7346	0.6072
12	0.1528	0.0003	163	0.7547	0.5869	N/A	N/A
13	0.4577	<2.2E‐16	146	0.9152	0.7186	0.8692	0.6822
14	0.2372	0.0006	92	0.6908	0.6321	0.8153	0.5545
15	0.1289	0.0347	107	0.4635	0.4881	N/A	N/A
16	0.0729	0.1006	156	0.5759	0.5365	N/A	N/A
18	0.1365	0.0001	212	0.6899	0.5739	N/A	N/A
Overall	0.4132	<2.2E‐16	1247	0.8008	0.6373	0.9500	0.6069

*R* ^2^ values from the fitted regression models of density state as a prediction of the spectral bands are shown for the individual fields and for the entire data sets, RGB and R_mod_ respectively. The random forest results show the ability of the data to predict the presence/absence (P/A) of black‐grass using the metrics area under a curve (AUC) and accuracy (ACC). A random forest model was also used to discriminate between high and very high (H/VH) levels of black‐grass using the same metrics.

## Results

### Explanatory power of UAS imagery

Examples of the different types of image that we used for building the models were produced by stitching the individual images together to form one analysable image (Fig. 1). Visual comparisons of Fig. 1A–C indicated that, visually at least, the variation was greater in the R_mod_ images. The grid overlay (Fig. 1C) represents the ground‐truthed data that we use for training the models.

**Figure 2:**
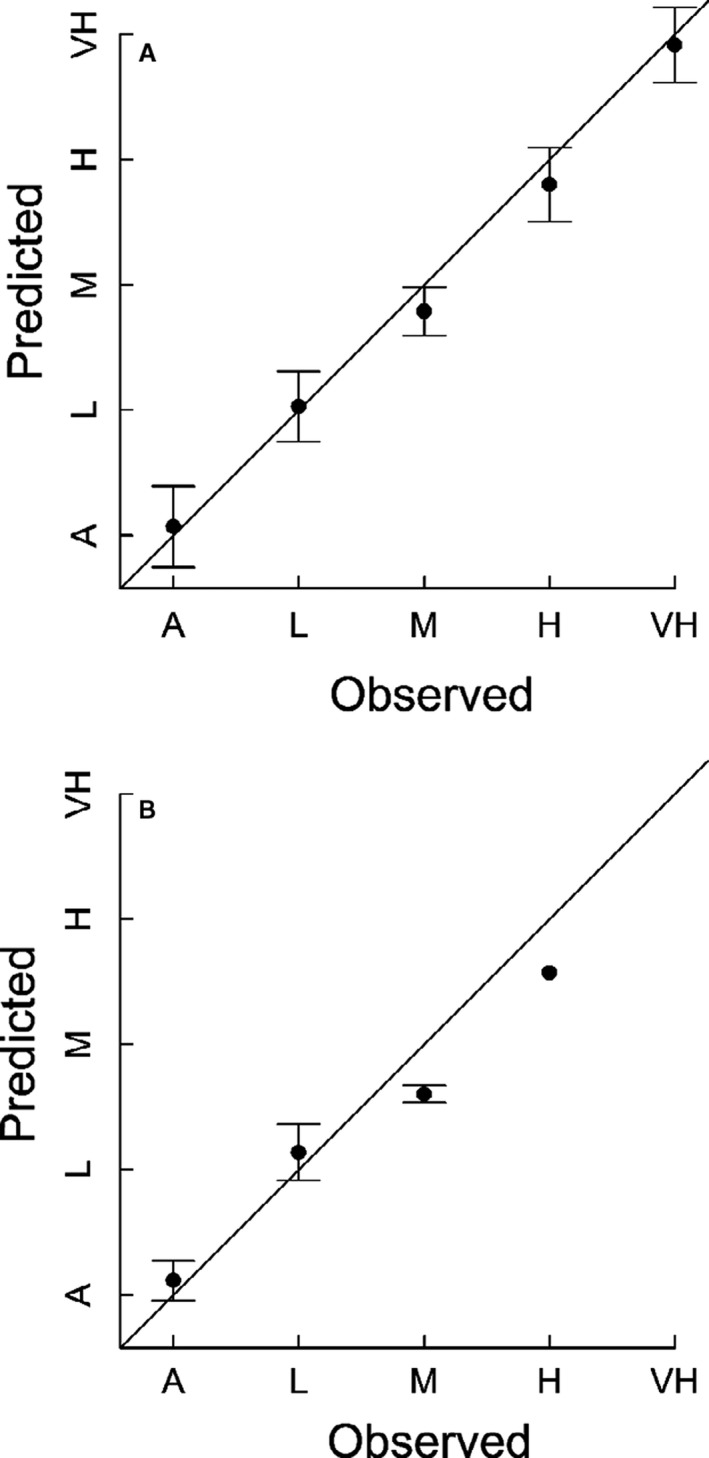
Fits of density state, against ground‐truthed observed data for the R_mod_ (A) and RGB (B) data sets respectively. The models were trained on 80% of the data and then tested against the remaining data for the predictions. Fits were generated from the linear regression models (see text for details).

We found that the variation within the images obtained from the UAS correlates with weed densities measured in the field. The accuracy of the method however varies with the data set used (Fig. 2) and between fields (Table 1). The linear model can characterise the relationship broadly across all the spectral bands. The RGB data performs well in some fields; however, overall the relationship between density states and the mean pixel values per 20 × 20 m plot is weaker, with a *R* ^2^ value of 0.29 compared with the R_mod_ *R* ^2^ value of 0.41 as evidenced in Fig. 2. Overall, we find that the R_mod_ data set has the largest *R* ^2^ value (0.41) when fitted to the entire data set, as well as the best performing individual field (0.46).

### Predictive ability

We used the random forest models described in Table 1 to test whether we could distinguish between areas with (presence) and without (absence) *A. myosuroides*. The RGB data set performed best overall (AUC = 0.88, Acc = 0.68). We also tested the ability of the random forest models to differentiate between areas of high and very high levels of *A. myosuroides*. Most fields being surveyed in the 2015 field season did not contain the full range (absent to very high) of *A. myosuroides* levels, thus we have relatively fewer data point to test this capability with. Nevertheless, the models still show a strong ability to distinguish between areas of high and very high levels of *A. myosuroides*. The R_mod_ data set has the highest AUC (0.95), but the corresponding accuracy (0.61) is the lowest of the data sets, this is an important factor to consider due to the lack of data and potential for false‐positives. The RGB data set shows a lower AUC (0.91) than R_mod_ but with a higher accuracy (0.87).

### Field‐to‐field predictions

**Figure 3:**
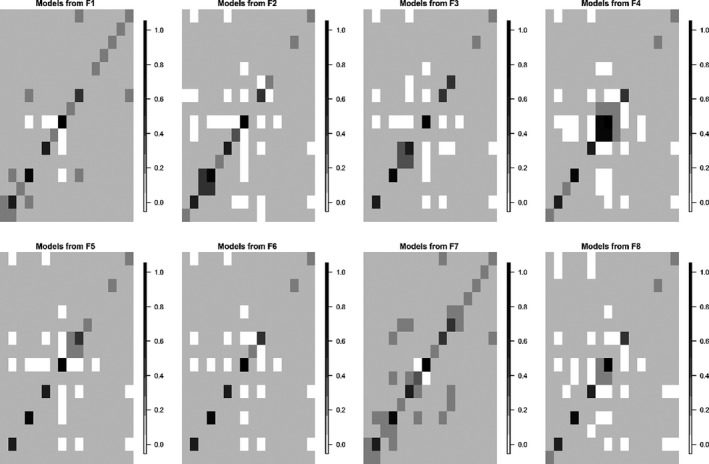
Heat map matrix, prediction correlation plots for a cubist model derived from field 1 to field 8 on the axis respectively from the R_mod_ data set. High correlation values indicate higher prediction accuracies of density states. The darker the cell, the higher correlation between the models predictions and the observed density state. White cells indicate NA's, these occur when the trained model did not predict a density state for every class that was present.

The heatmaps in Fig. 3 summarise the overall analysis of inter‐field predictions. Each cell in the respective matrices represents a correlation coefficient of the observed density states and predicted states from the cubist models that have been trained on only one field's worth of data. The results from this analysis are mixed. Although some correlations are relatively high, the average correlation for all the models was relatively weak (0.34). This suggests that the cubist models were locally over‐fitting the relationship between density state and the spectral signal, resulting in poorer field‐to‐field transferability of the models.

## Discussion

Our main finding was that aerial images collected with a low‐cost UAS (<€1000) have the potential to be used to map populations of *A. myosuroides*. However, our results indicate that if this technology is to be applied at a large scale in an automated way, then there are several issues that need to be addressed. Secondly, our analyses of within‐field variation using simple statistical models show that it is possible in principle to capture the variation in weed densities. However, models developed in one field rarely perform well when applied elsewhere, indicating that locally they were over‐fitting the relationship between density state and the spectral signal. This means that currently the interpretation of such imagery is limited without supporting ground‐truthed data; the ultimate objective of our research is to be able to generate estimates of densities from imagery without the need for detailed ecological surveys. Year on year transferability is currently being assessed. We have highlighted that there are challenges in generating robust predictive models that relate variation within images to weed densities within fields, yet are applicable across multiple sites. Our work has revealed areas that need to be streamlined for the methodology to become more of a tool for management applications.

### Choice of spectral frequency

We found the most informative spectral frequency to be red‐edge (R_mod_). Of the sets of spectral bands we tested, R_mod_ captured the relationships between the pixel values and ground observations of *A. myosuroides* density state most accurately. There is an extensive literature on the uses of indices such as NDVI (Normalized Difference Vegetation Index) and EVI (Enhanced vegetation index) with the use of satellite data (Xie *et al*., 2008; Pettorelli, 2013). These indices have been used in conjunction with some UAS studies, although they have mainly been proof of concept, due to technical limitations and their focus on small scale, high value crops, such as vines (Turner *et al*., 2011; Primicerio *et al*., 2012), while rarely addressing ecological monitoring problems.

### Choice of analysis

Torres‐Sánchez *et al*. (2014) used UAS to map weeds in an agricultural setting, although they primarily focused on capturing the aerial images in early season for the crops. This means that there are discernible rows of the crop from time of planting. Object‐based image analysis (OBIA) has been the most commonly used methodology to detect weeds when studying this type of data (Pena *et al*., 2013). This approach is useful in the management of weeds in the early part of the season and has applications informing in‐season decisions. Late in the season, rows are not discernible in crops like cereals, which have dense overlapping canopies when mature and hence these approaches are likely to be less useful.

Our approach focused on late‐season imaging of the crops. This reflects in part the purpose of our original modelling framework (Freckleton *et al*., 2011; Queenborough *et al*., 2011), which was to parameterise ecological models to project future weed densities. Monitoring late‐season weed numbers should give insight into where the weeds will emerge next year due to seed set. Rather than inform current management practices, such information can be used to make decisions in subsequent seasons, such as patch‐spraying (Audsley, 1993) or variable‐sowing densities (Chauhan *et al*., 2011). The two approaches (late season versus early season monitoring) can work in conjunction with one another. One useful application of combining approaches would be to check the effectiveness of the management decisions previously made. However, the technical challenges of monitoring at different times are likely to be somewhat different.

### Automation

To be of general use in both research and management, the process should be as automated as possible, requiring minimal interventions by the analyst. However, this requires that several key problems are solved. Most notably, as indicated by our results, images vary from field‐to‐field, so that the relationship between density and image intensity is not fully transferable from one field to another. Increasing the comparability of images is thus a key priority, for example through accounting for variable lighting and by standardising spectra.

A key assumption of image interpretation is that we are detecting *A. myosuroides*. In the current analysis, we have specifically focused on *A. myosuroides* and we have extensive ground‐truth data to test the ability of imagery to detect this. In an automated system, we would ideally be able to proceed with minimal ground‐truth data. The extent to which variation resulting from, for example, poor crop establishment, other weeds or disease, rather than the presence of *A. myosuroides* is unknown. In terms of in‐field management, this may not matter; output could still be informative to the farmer and agronomist. Variation in image intensity within‐field maps would act as ‘signposts’ to areas of the field that we have shown to be different from the normal crop. They would then be able to field walk‐specific locations. This means that ground‐truthing of the maps is still required to detect what the actual causes of the variation in the field are and automation would not be achieved. However, for the purposes of wide‐scale mapping for larger areas or as a research tool, it will be important to examine how distinct factors can be distinguished. For example, yellowing of a wheat crop owing to disease such as *Puccinia striiformis* f.sp.* tritici* (yellow rust) (Moshou *et al*., 2004) should be distinguishable from *A. myosuroides* based on spectral characteristics.

When looking at ways to automate data collection, one important issue is setting a threshold for the detection of soil. In our current methodology, we manually set the threshold for each field by viewing the histogram of the pixel intensities in imageJ and then manually removing the pixels that fell below a cut‐off value. This analytical step could be improved using several approaches. For example, an OBIA system could be used to detect tramlines and then set an applicable boundary either side of each track to mask all the soil pixels from the analysis. Alternatively, a clustering‐based image thresholding technique such Otsu's method could be applied (Shorter & Kasparis, 2009). The challenge is to determine how such an algorithm flexibly accounts for differences in soil colour between fields.

We find similarities between this work and that of Dvořák *et al*. (2015), in that they used UAS to map alien invasive species using pixel‐based classification. They also highlighted the challenges of unstable scene illumination, an issue that individual field analysis presented. By compiling all the respective grid square data into one data set, rather than the current field‐by‐field data sets, we hope to mitigate some of the variation introduced by the unstable lighting conditions. New sensor technologies to combat issues such as this are constantly being released; one example is the recent announcement of an integrated imaging system and sun irradiance sense from MicaSense called Sequoia.

### Limitations and future work

The limitations of this technology and methodology are that it is not completely independent of field walking to gather the ground‐truthed data. The statistical methods used here are relatively unsophisticated and are potentially not utilising all the features of the current data. The current feature design of only using the mean pixel value for each 20 × 20 m grid is rudimentary, so in further studies, we would include more features, such as spatial correlation and field management histories. Nonetheless, this methodology has potential to amplify the work of field surveying, allowing data to be gathered on a scale that is currently unachievable for a small team. A team of field surveyors can produce a more accurate map of *A. myosuroides* than our current UAS method. Indeed, such data can be entered onto a computer at the time of mapping and a field‐scale map generated that, if an Internet connection is available, can be immediately uploaded and distributed. In contrast, the analysis of UAS‐derived data requires several steps, including image stitching that can take several hours of computational time.

The advantages of using UAS are in terms of scale and a minimal analysis needed to assess *A. myosuroides* levels. There is generally expected to be a trade‐off between extent of measurement and precision, and this is true for arable weeds (Marshall, 1988). As we have shown recently, relatively coarse data can be extremely valuable for measuring weed populations, if they are available at sufficiently large scales (Queenborough *et al*., 2011; Freckleton *et al*. in revision). In the case of imagery from UAS, it is potentially possible to generate finer‐scale maps than can be generated using techniques such as the field walking methods of Queenborough *et al*. (2011) and at greater speed. Hence, there is the potential for UAS‐derived imagery to allow a step change in the extent and accuracy of data collection.

There has been work to integrate the use of UAS into Site Specific Weed Management (SSWM) as the UAS allows for efficient and repeatable collection of spatial data (Torres‐Sánchez *et al*., 2013). Their study set out to describe the technical specifications and configuration of a UAS that can be used in SSWM. Farmers already use *A. myosuroides* maps, such as those generated by our ground‐truthed data, to implement variable seed‐rate planting (Helen Hicks pers. obs.). This allows farmers to plant crops at a higher seed density in areas known to have had high weed burdens in the previous year. The aim of this is to outcompete *A. myosuroides* in the early stages of germination, resulting in less *A. myosuroides* setting seed (Timmermann *et al*., 2003). The development of UAS‐based weed mapping systems has the potential to provide weed maps more quickly and at a lower cost to the farmer. It is also important to understand that this work is tackling one of the most challenging issues in the field of weed mapping, identifying one mature grass within another mature grass, and therefore, there may be an upper limit in prediction accuracy.

In addition to developing technology that could be used for informing agronomic decision‐making, development of these data collection and processing techniques are important for research. A major factor in collection of population monitoring data is the difficulty in collecting enough data for model development within time and budget constraints *(*Bryson *et al*., 2014). The new methodology developed here, using UAS to collect highly detailed images of populations and building predictive statistical models, could potentially be applied to many population monitoring studies, such as rangeland and invasive weed mapping (Rango *et al*., 2009; Hung *et al*., 2014). However, our results indicate that there are obstacles to be overcome particularly if we are to avoid extensive ground‐truthing and be able to readily apply such methodology to different fields and farms.
